# An In Vivo Definition of Brain Histamine Dynamics Reveals Critical Neuromodulatory Roles for This Elusive Messenger

**DOI:** 10.3390/ijms232314862

**Published:** 2022-11-28

**Authors:** Shane N. Berger, Beatrice Baumberger, Srimal Samaranayake, Melinda Hersey, Sergio Mena, Ian Bain, William Duncan, Michael C. Reed, H. Frederik Nijhout, Janet Best, Parastoo Hashemi

**Affiliations:** 1 Department of Chemistry and Biochemistry, University of South Carolina, Columbia, SC 29208, USA; 2 Department of Bioengineering, Imperial College London, London SW7 2AZ, UK; 3 Department of Physiology, Pharmacology & Neuroscience, University of South Carolina School of Medicine, Columbia, SC 29209, USA; 4 Department of Mathematics, Montana State University, Bozeman, MT 59717, USA; 5 Department of Mathematics, Duke University, Durham, NC 27710, USA; 6 Department of Biology, Duke University, Durham, NC 27708, USA; 7 Department of Mathematics, Ohio State University, Columbus, OH 43210, USA

**Keywords:** voltammetry, inflammation, sex differences, FSCV, thioperamide

## Abstract

Histamine is well known for mediating peripheral inflammation; however, this amine is also found in high concentrations in the brain where its roles are much less known. In vivo chemical dynamics are difficult to measure, thus fundamental aspects of histamine’s neurochemistry remain undefined. In this work, we undertake the first in-depth characterization of real time in vivo histamine dynamics using fast electrochemical tools. We find that histamine release is sensitive to pharmacological manipulation at the level of synthesis, packaging, autoreceptors and metabolism. We find two breakthrough aspects of histamine modulation. First, differences in H3 receptor regulation between sexes show that histamine release in female mice is much more tightly regulated than in male mice under H3 or inflammatory drug challenge. We hypothesize that this finding may contribute to hormone-mediated neuroprotection mechanisms in female mice. Second, a high dose of a commonly available antihistamine, the H1 receptor inverse agonist diphenhydramine, rapidly decreases serotonin levels. This finding highlights the sheer significance of pharmaceuticals on neuromodulation. Our study opens the path to better understanding and treating histamine related disorders of the brain (such as neuroinflammation), emphasizing that sex and modulation (of serotonin) are critical factors to consider when studying/designing new histamine targeting therapeutics.

## 1. Introduction

Histamine is a biological amine best known for mediating peripheral inflammation. Bodily histamine is synthesized in immune cells including mast cells [1,2] and T-cells [3]. Histamine is also found in the brain in microglia [4,5] and neurons [6]. The histaminergic cell bodies reside in the tuberomammillary nucleus (TMN) and innervate the entire brain [6,7,8,9], where the primary action of this messenger is thought to be neuromodulatory [4,10,11]. Histamine has been shown to automodulate and to modulate serotonin, dopamine, acetylcholine, glutamate and GABA [12,13,14,15,16].

An elegant body of work has revealed the critical importance of histamine modulation on the brain’s processes [17,18,19]. However, because dynamic histamine chemistry is very difficult to measure, several fundamental aspects of the mechanisms that control the extracellular and modulatory behavior of this messenger remain undefined. Central nervous system histamine has been previously studied using brain homogenates [20], cellular supernatant assays [21], in vivo microdialysis coupled to high-performance liquid chromatography [22,23], and electrophysiology [22].

We recently developed a voltammetric method that can measure histamine in real time in vivo [24]. The method, fast-scan cyclic voltammetry (FSCV), facilitates sub-second evoked histamine measurements in the TMN from implanted electrodes with minimal spatial footprint, carbon fiber microelectrodes (CFMs). We further used this method to measure histamine and serotonin simultaneously in vivo and discovered that histamine instantaneously inhibits serotonin release via (inhibitory) histamine 3 (H3) heteroreceptors [13,25]. In follow up work, we showed that acute peripheral inflammation in mice increased brain histamine and showed real-time evidence for active histamine reuptake via organic cation transporters (OCTs) [25].

Having understood from this previous work that histamine plays critical roles in neuromodulation (in particular, with respect to serotonin), we here sought to create a detailed framework of in vivo histamine signaling. This work takes an in-depth approach to characterizing in vivo histamine dynamics in male and female mice by pharmacologically targeting histamine synthesis, packaging, receptor control of release, reuptake, and metabolism. Our work constitutes the first thorough characterization of fast histamine transmission in vivo and presents two meaningful, breakthrough aspects of histamine modulation; the differences in H_3_ regulation between the sexes and H_1_ modulation of serotonin dynamics. These findings will enable a better understanding of the nuances of histamine related disorders of the brain (such as neuroinflammation), highlighting the fact that sex and serotonin modulation are critical considerations for histamine targeting therapeutics.

## 2. Results

### 2.1. Evoked Histamine in Male and Female Mice

**Figure 1:**
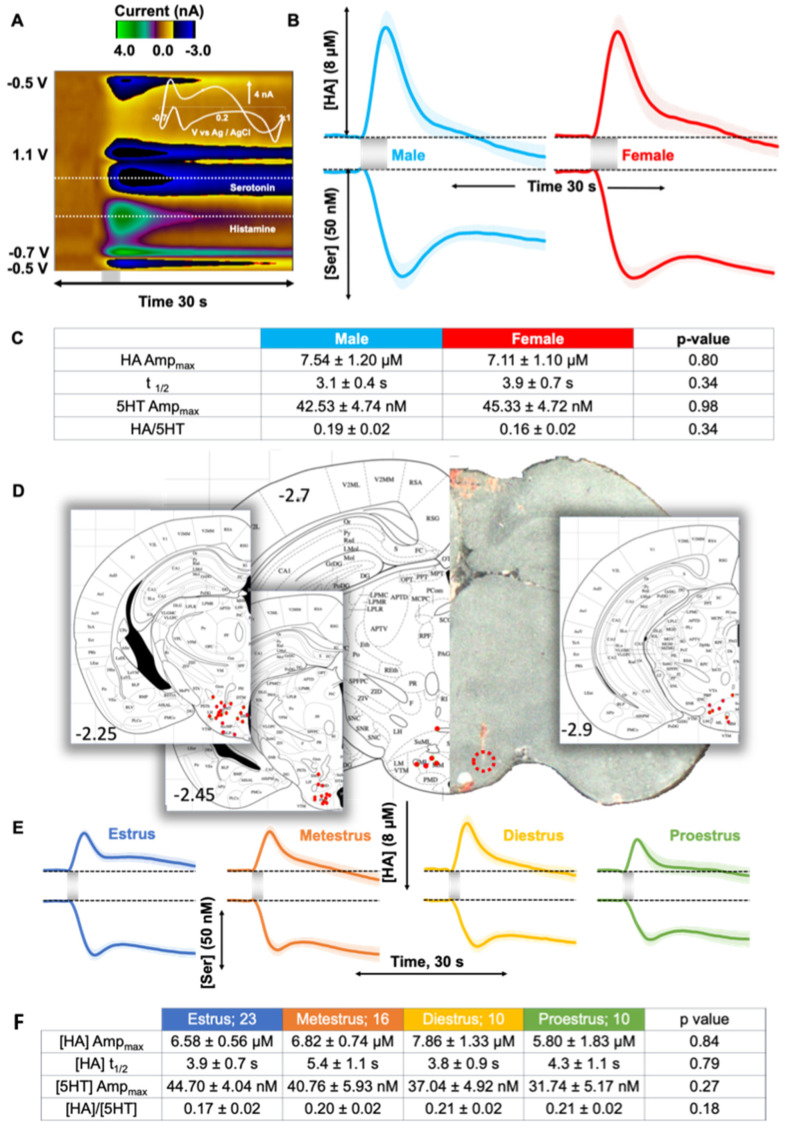
(**A**) Representative color plot of the mouse TMN upon MFB stimulation. Inset in the top right corner is the characteristic CV with peaks occurring around 0.2 V for histamine and 0.7 V for serotonin oxidation. The concentration vs. time traces for the release of histamine and inhibition of serotonin are shown for (**B**) male (blue) and female (red) mice. Electrical stimulation (2 s) is represented by the grey bars. (**C**) Tabulated values of metrics of experimental data in male and female mice. (**D**) Placement of the CFMs in all experiments in this paper in the lateral hypothalamus. (**E**) Evoked histamine release and serotonin inhibition for female mice in estrous (*n* = 23), metestrus (*n* = 16), diestrus (*n* = 10), and proestrus (*n* = 10) stages. The shaded grey bar represents the 2 s electrical stimulation. (**F**) Tabulated values of metrics of experimental data in female mice in different stages of the estrous cycle.

We first developed a method to measure histamine in vivo with voltammetry in 2015, ref. [24] and utilized male mice as proof of principle. In follow up work we showed simultaneous inhibition of serotonin, via the H3 receptors, when histamine was electrically evoked [13]. Here, we repeated those same experiments, where the MFB was stimulated and the resultant, evoked histamine was measured in the posterior hypothalamus in both male and female mouse cohorts (Figure 1). Figure 1A is a representative color plot with a CV inset in the top right corner. Interpretation of these color plots is described in detail elsewhere [26], briefly the FSCV potential ramp is on the y axis, the time of acquisition on the x axis and the current is in a color blind-friendly false color scale. The averaged concentration vs. time traces are shown in Figure 1B where the stimulated histamine release in male mice was 7.5 ± 1.2 µM and 7.1 ± 1.1 µM in females. The magnitude of serotonin inhibition was 42.5 ± 4.7 nM in male mice and 45.3 ± 4.7 nM in females.

Tabulated in Figure 1C are the histamine release and serotonin inhibition amplitudes, the rate of decay of the stimulated histamine release (t_1/2_: male: 3.1 ± 0.4 s; female: 3.9 ± 0.7 s; *p* = 0.34) and the ratio of peak histamine release to peak serotonin inhibition. The sample size was equal for male and female mice at *n* = 20. There were no statistical differences in any of these metrics between male and female mice. Figure 1D shows the placement of the CFMs in all experiments in this paper in the lateral hypothalamus.

We next evaluated the effect of estrous cycle stage on the signal in female mice. Figure 1E shows averaged signals in female mice throughout estrus, metestrus, diestrus, and proestrus stages (verified via vaginal lavage).

We found no significant difference in the evoked histamine amplitude (Amp_max_: estrus (blue; *n* = 23): 6.6 ± 0.6 µM; metestrus (orange; *n* = 16): 6.8 ± 0.7 µM; diestrus (yellow; *n* = 10): 7.9 ± 1.3 µM; proestrus (green; *n* = 10): 5.8 ± 1.8 µM; *p* = 0.84 Kruskal–Wallis H-test) or t_1/2_ (estrus: 3.9 ± 0.7 s; metestrus: 5.4 ± 1.1 s; diestrus: 3.8 ± 0.9 s; proestrus: 4.3 ± 1.1 s; *p* = 0.79 Kruskal–Wallis H-test) of reuptake curve across estrous stages. The peak serotonin inhibition was also not significantly different and shown across cycle stages (Amp_max_: estrus (blue; *n* = 23): 44.7 ± 4.0 nM; metestrus (orange; *n* = 16): 40.8 ± 5.9 nM; diestrus (yellow; *n* = 10): 37.0 ± 4.9 nM; proestrus (green; *n* = 10): 31.7 ± 5.2 nM; *p* = 0.27 Kruskal–Wallis H test). These data are tabulated, with the addition of the histamine/serotonin amplitude ratio in the table in Figure 1F. There were no significant differences in any of these metrics.

### 2.2. Targeting Histamine Packaging, Synthesis and Metabolism

**Figure 2:**
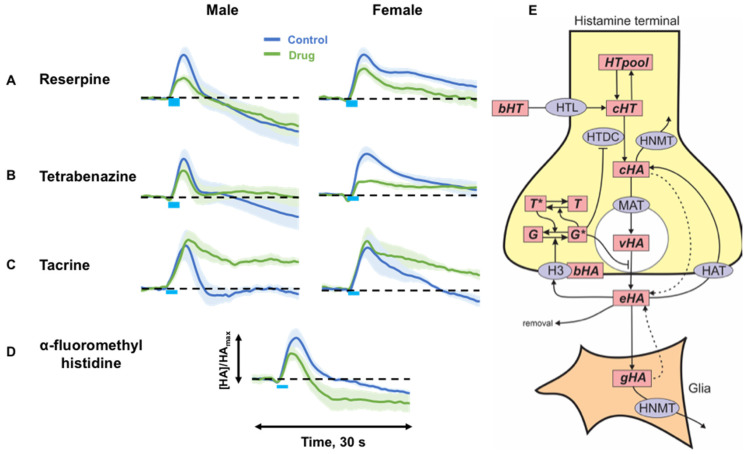
(**A–D**) Averaged concentration vs. time traces for control (blue) and post-drug (green) evoked histamine. The drug and mouse’s sex are listed. For α-fluoromethylhistidine male and female traces were averaged (**E**) Schematic representation of basic histamine metabolism, abbreviations are: bHT, blood histidine; cHT, cytosolic histidine; HTpool, the histidine pool; cHA, cytosolic histamine; vHA, vesicular histamine; eHA, extracellular histamine, gHA, glial cell histamine; bHA, the concentration of bound autoreceptors; G and G∗, the inactive and active G-protein subunit; T and T∗, the inactive and active RGS protein; HTL, the histidine transporter; HTDC, histidine decarboxylase; HNMT, histamine methyltransferase; HAT, the putative HA transporter; H3, histamine autoreceptor.

Next, we investigated the mechanisms that control extracellular histamine levels in vivo. Figure 2 shows average evoked histamine (normalized to maximum control level) before (control, blue) and after (green) drug manipulation (*n* = 5 to 6 each).

We studied histamine packaging using two VMAT inhibitors with different affinities for VMAT1 and VMAT2 (Figure 2A,B) (reserpine: 10 mg kg^−1^ and tetrabenazine (TBZ): 10 mg kg^−1^). 60 min following reserpine, a significant decrease in evoked histamine was observed in both male (vehicle: 5.4 ± 0.6 µM; reserpine: 2.7 ± 0.5 µM; *p* = 0.009 paired *t*-test) and female mice (vehicle: 9.2 ± 1.2 µM; reserpine: 6.5 ± 1.0 µM; *p* = 0.016 paired *t*-test). There was no change in the rate of reuptake of histamine for either sex (male: t_1/2_: control: 2.4 ± 0.7 s; reserpine: 2.8 ± 0.7 s; *p* = 0.51 paired *t*-test) (female: t_1/2_: control: 6.3 ± 2.5 s; reserpine: 4.4 ± 1.6 s; *p* = 0.23 paired *t*-test). 60 min after mice received TBZ, a significant decrease in evoked histamine was observed for both sexes (male vehicle: 7.1 ± 1.4 µM; TBZ: 5.5 ± 1.4 µM; *p* = 0.023 paired *t*-test) (female vehicle: 7.6 ± 0.8 µM; TBZ: 3.8 ± 0.4 µM; *p* = 0.008 paired *t*-test). We next targeted synthesis and metabolism by using tacrine (Figure 2C), an N-methyltransferase inhibitor, and α-fluoromethylhistidine (FMH) (Figure 2D), an L-histidine decarboxylase inhibitor. Administration of tacrine to male mice resulted in no change in histamine amplitude (control: 9.6 ± 0.9 µM; tacrine: 9.4 ± 1.2 µM; *p* = 0.92 paired *t*-test) but slowed clearance of histamine from the extracellular space (t_1/2_: control: 2.8 ± 0.8 s; tacrine: 6.0 ± 0.7 s; *p* = 0.025 paired *t*-test). In female mice there was also no amplitude change following tacrine (control: 7.0 ± 1.8 µM; tacrine: 7.7 ± 1.9 µM; *p* = 0.22 paired *t*-test) and a similar, but non-significant reuptake change (t_1/2_: control: 4.8 ± 1.4 s; tacrine: 4.9 ± 2.4 s; *p* = 0.095 paired *t*-test).

FMH availability was severely limited, thus we combined the male and female responses into one grouping for the next experiment. 60 min following this inhibition of histamine synthesis, a significant decrease in stimulated histamine was observed (control: 8.6 ± 1.9 µM; post-FMH: 5.8 ± 1.2 µM; *p* = 0.038 paired *t*-test). There was no change in histamine clearance ((t_1/2_: control: 2.4 ± 0.2 s; FMH: 2.3 ± 0.4 s; *p* = 0.82 paired *t*-test). In none of these experiments were the male and female responses substantially different from each other. Figure 2E shows our proposed model of histamine synthesis, packaging, reuptake and metabolism.

### 2.3. H3R Autoreceptor Control in Male and Female Mice

**Figure 3:**
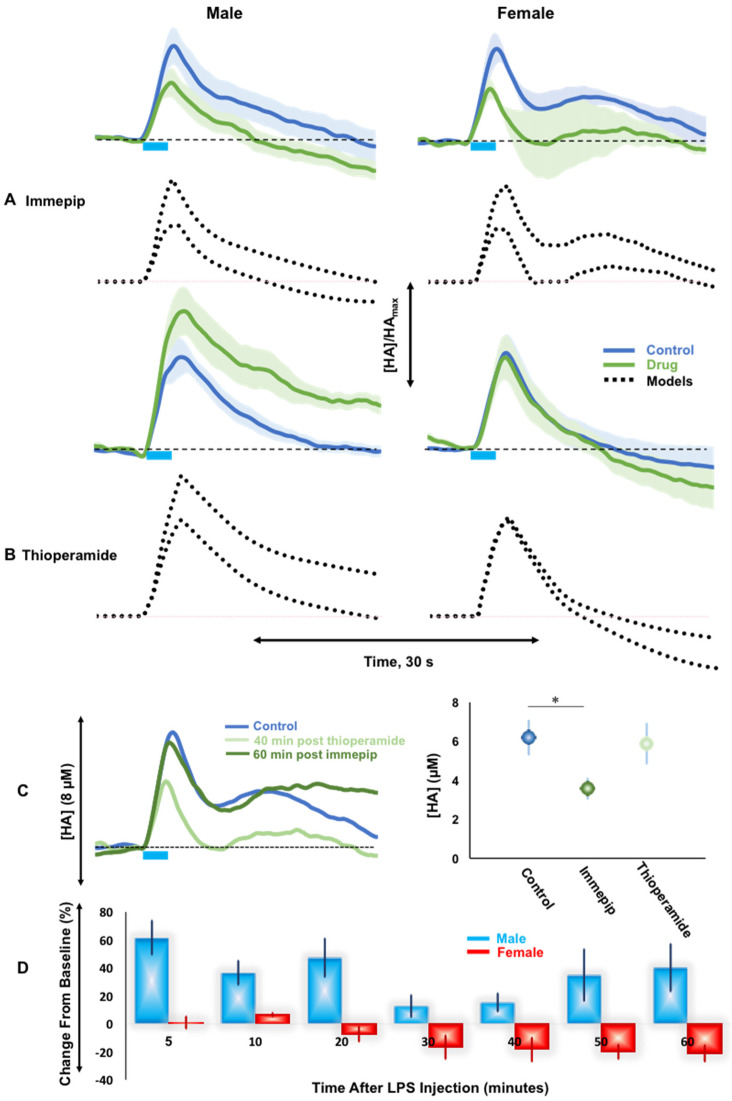
(**A**,**B**) The drug and mouse’s sex are listed. Concentration vs. time traces for control (blue) and post-drug (green) evoked histamine. The mathematical model results are in black dotted lines. (**C**) [HA] vs. time profiles of evoked histamine for control (*n* = 5, blue), 60 min following immepip (*n* = 5, dark green), and 40 min following thioperamide after 60 min immepip (*n* = 4, light green). Max amplitude of evoked histamine for control (blue), 60 min immepip (dark green), and 40 min following thioperamide after initial 60 min immepip (light green). Significance between two points was taken as *p* < 0.05 (**D**) Male vs. female % difference (vs. control) in evoked histamine release with time after LPS injection. * = *p* < 0.05.

Next, we investigated autoreceptor control of histamine release in male and female mice via an H3R agonist, immepip (5 mg kg^−1^), and an H3R antagonist, thioperamide (20 mg kg^−1^) in Figure 3A–C. Male and female mice respond similarly to H3R agonism with an overall decrease in maximum amplitude (male control: 7.7 ± 1.6 µM; post-drug: 4.8 ± 1.6 µM; *p* = 0.024; female control: 6.2 ± 0.9 µM; post-drug: 3.6 ± 0.5 µM; *p* = 0.005 paired *t*-test) and no change in histamine clearance (male t_1/2_: control: 4.5 ± 1.7 s; post-drug: 2.9 ± 0.6 s; *p* = 0.4; female t_1/2_: control: 5.6 ± 1.8 s; post-drug: 5.6 ± 2.6 s; *p* = 1 paired *t*-tests).

We mathematically treated these responses with a previously developed model (see methods) and show the fits to experimental data in Figure 3A,B. The major model parameter that was changed to fit the post-immepip data in both male and female mice was the strength of the H3 autoreceptor. For the male and female control curves, this parameter was set to 0.9. We obtained the post-drug curve by increasing the strength of the autoreceptor to 1.9 in both males and females.

Thioperamide administration caused a significant increase in evoked hypothalamic histamine in male mice (control: 8.8 ± 1.4 µM; post-drug: 12.1 ± 1.8 µM; *p* = 0.046) and no significant change in histamine clearance (control: 4.5 ± 1.7 s; post-drug: 9.9 ± 2.2 s; *p* = 0.051). However, in the female mice, no change in amplitude (control: 7.4 ± 1.4 µM; post-drug: 7.0 ± 1.7 µM; *p* = 0.39) or rate of reuptake (t1/2: control: 4.2 ± 1.6 s; post-drug: 3.9 ± 1.3 s; *p* = 0.48) was observed following the same dose of thioperamide.

When we modeled these responses, in male mice we obtained the post-drug curve by decreasing the strength of the autoreceptor from 0.9 to 0. In female mice however, thioperamide did not change the response, thus the autoreceptor strength in the model remained constant.

To test whether this significant finding between male and female mice was a consequence of female mice simply not responding to thioperamide, we did a further experiment where we first agonized the H3 receptor (via immepip (5 mg kg^−1^) 60 min), and then antagonized the H3 receptor in the same, female mice with thioperamide (20 mg kg^−1^).

Figure 3C shows the histamine vs. time profiles of control (blue), 60 min post-immepip (dark green) followed by 40 min post-thioperamide (light green). Following a significant decrease from control with immepip, we found that thioperamide restored stimulated histamine only to control levels (immepip: 3.6 ± 0.5 µM; post-immepip-thioperamide: 5.9 ± 1.1 µM; *p* = 0.13); the evoked histamine in female mice did not increase above control values in this (control: 6.2 ± 0.9 µM; post-immepip-thioperamide: 5.9 ± 1.1 µM; *p* = 0.83 paired *t*-test), or any of the other experiments in this work.

In previous work, we showed that lipopolysaccharide (LPS) injection in mice increased histamine release levels, likely as a consequence of inflammation [25]. Here, we repeated this finding first in male mice, represented by the blue bars in Figure 3D. This chart shows % difference (w.r.t. to each sex’s control) in evoked histamine release, averaged between animals, with time after LPS injection (i.p). In male mice, LPS injection increased histamine release rapidly after 5 min (post hoc *t*-test, 61.6 ± 12.1% increase, *p* < 0.01). We performed the same experiments in female mice where LPS failed to significantly increase evoked histamine 5 min (post hoc *t*-test, 0.5 ± 4.6% increase, *p* = 1.00) or 10 min after injection (post hoc *t*-test, 6.6 ± 1.4% increase, *p* = 0.40) and trends for a decrease in histamine amplitude 60 min after injection (post hoc *t*-test, -21.1 ± 5.8% increase, *p* = 0.18). Importantly, again in female mice, evoked histamine did not substantially increase above a control threshold. A two-way analysis of variance (ANOVA) (with effects of sex and time after injection) showed that there was a statistically significant effect of the interference of the two factors (F = 3.26, *p* < 0.01) showing that LPS injection is different between the sexes. A Tukey–Kramer post hoc analysis of the individual groups was then performed.

### 2.4. Histamine Post-Synaptic Receptor Pharmacology

**Figure 4:**
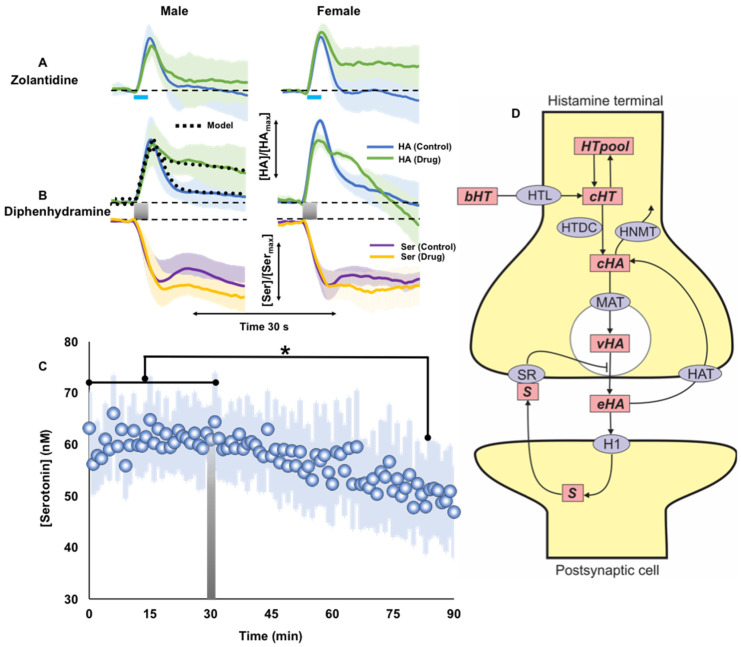
(**A**,**B**) The drug and mouse’s sex are listed. Concentration vs. time traces for control (blue) and post-drug (green) evoked histamine, and control (purple) and post-drug (orange) inhibited serotonin. The light blue bar and the shaded grey bar represent the 2 s electrical stimulation. The mathematical model’s simulations are in black dotted lines. (**C**) Extracellular ambient serotonin levels in the CA2 region of the hippocampus measured once a minute after a large DPH dose (gray bar) (*n* = 5) * is the first data point where the signal is significantly different from control. (**D**) Schematic diagram of histamine transmission via the H1R. Abbreviations: bHT, blood histidine; cHT, cytosolic histidine; HTpool, the histidine pool; cHA, cytosolic histamine; vHA, vesicular histamine; eHA, extracellular histamine; HTL; HTDC, histidine decarboxylase; HNMT, histamine methyltransferase; HAT, the HA transporter; HTL, the histidine transporter, H1, H1R; S, inhibitory retrograde messenger. * = *p* < 0.05.

We next investigated post-synaptic H1 and H2 receptors. Figure 4A shows response of male and female mice to zolantidine (10 mg kg^−1^), an H2 antagonist. No significant change in evoked histamine release was seen in male and female mice given zolantidine (male control: 5.8 ± 0.6 µM; post-drug: 4.9 ± 0.6 µM; *p* = 0.33 paired *t*-test, female control: 5.4 ± 0.7 µM; post-drug: 5.5 ± 0.9 µM; *p* = 0.96 paired *t*-test). Clearance profile was also not significantly different (male t1/2: control: 4.2 ± 1.3 s; post-drug: 3.2 ± 1.0 s; *p* = 0.46 paired *t*-test, female t1/2: control: 2.7 ± 0.7 s; post-drug: 3.8 ± 1.6 s; *p* = 0.59 paired *t*-test).

Figure 4B shows the response to diphenhydramine (DPH) (20 mg kg^−1^), an H1 antagonist. DPH did not significantly change the release amplitude in either sex (male control: 9.2 ± 3.1 µM; post-drug: 9.4 ± 2.7 µM; *p* = 0.83 paired *t*-test, female control: 6.8 ± 0.2 µM; post-drug: 5.3 ± 0.6 µM; *p* = 0.08 paired *t*-test). In both sexes a significant decrease in clearance occurred between 50-60 min post DPH (male t1/2 control: 2.7 ± 0.4 s; post-drug: 7.2 ± 1.1 s; *p* = 0.01 paired *t*-test, female t1/2: control: 3.3 ± 0.8 s; post-drug: 12.0 ± 2.6 s; *p* = 0.04 paired *t*-test). To understand the mechanism of this decreased clearance, we modeled the data in male mice. To fit the data, the model required incorporation of a strong inhibition of histamine release from t = 9 s to t = 15 s (control curve), indicating an ongoing inhibitory stimulus on the presynaptic cell in response to H1 activation (substance S in Figure 4D). DPH blocks the H1Rs, so in the presence of DPH the histamine signal to the post-synaptic cell is reduced, we thus assumed in the presence of DPH that this inhibition of HA release was removed. When we did this, we obtained the post-drug curve in Figure 4B, lending strong support to the hypothesis.

This slowed clearance is a finding of interest since DPH is a common over the counter antihistamine drug and we know that histamine inhibits serotonin [13,17]. We therefore studied the histaminergic inhibition of serotonin in these same mice, shown in Figure 4B. The inhibition profile of serotonin was not statistically significant in either case, however there seemed to be a trend towards increased inhibition after DPH. Therefore, we formally tested how a large DPH dose may affect extracellular serotonin levels. We measured extracellular ambient serotonin levels in the CA2 region of the hippocampus once a minute with fast scan controlled adsorption voltammetry (FSCAV) (Figure 4C) and saw a significant decrease in serotonin with respect to a large DPH dose (50 mg kg^−1^) (*n* = 5).

To certify this drop in serotonin levels, a two-way ANOVA (with effects of treatment and mice) was performed on the basal data. A significant effect of the treatment was found, (F = 4.85, *p* < 0.01). Tukey–Kramer multiple comparisons *t*-test identified that the first significant decrease in serotonin respect to the control state was 42 min after DPH injection (60.7 ± 1.3 vs. 50.3 ± 8.4, *p* = 0.03). An analysis of covariance (ANCOVA) was then performed to compare the slopes of the average control state and after DPH injection (F = 31.88, *p* < 0.01). The multiple comparisons *t*-test showed a significant decrease in the slope of the curve after DPH injection (0.05 nM/min vs. -0.21 nM/min, *p* < 0.01). Figure 4D shows our proposed model of post synaptic histamine signaling.

## 3. Discussion

### 3.1. Control Evoked Histamine Does Not Vary between Sexes

In this study we set out to investigate the machinery of the central histaminergic systems in mice. Under control conditions, electrically stimulated histamine release and reuptake was measured with FSCV in the TMN. We did not find any significant differences in the release and reuptake of hypothalamic histamine between male and female mice. This finding agrees with our own previous work that compared hippocampal serotonin between sexes and found no statistical differences [27]. Our results differ from some literature reports that suggested histamine turnover and histamine cerebrospinal fluid concentration are higher in females [28] or show lowered histamine release from tissue slice preparations in females [21]. These previous studies were ex vivo and in vitro, which by their nature involve observing the extrinsic system. Our in vivo model keeps innate brain circuitry intact, thus these previous studies may indicate that sex differences can be found in subtler, circuit or molecular levels (vide infra). Additionally, our data are not normalized, highlighting the high level of conservation in neurochemical regulatory mechanisms across individual mice.

There is an intrinsic belief that there may be neurochemical differences between the different stages of the estrous cycle that has limited the use of females in research [29]. Due to histamine’s potential role in neuroinflammation, this notion is important to explore; the extent of immune reactivity has been thought to depend on the different stages of the estrous cycle [30]. Therefore, we compared evoked histamine in female mice during different stages of the estrous cycle and found that histamine was not significantly different throughout. This finding is not surprising given our prior experience with measuring neurotransmitters with FSCV where we have had to employ aggressive pharmacological means to affect a significant but small change from homeostasis. Additionally, we saw no difference in serotonin releases, clearance, or extracellular levels in female mice during different stages of the estrous cycle [27].

Our histamine measurements are in a specifically targeted region of the posterior hypothalamus where we detect both evoked histamine and the resulting inhibition of serotonin. This has been shown to be an H3R mediated process by our lab and others [13,17,31]. After confirming no statistical differences in evoked histamine between male and female mice and throughout the estrous cycle, we analyzed the level of serotonin inhibition resulting from histamine release in the same mice. Here, also, the inhibition of serotonin is not different between males and females, and the ratio of maximum release to peak inhibition does not differ. Throughout the estrous cycle serotonin inhibition did not vary significantly.

Thus, from these data it is clear that under control conditions, histamine signaling is conserved between male and female mice. We next asked whether histamine release was dependent on the mechanics of histamine synthesis, packaging and metabolism.

### 3.2. Histamine Release Is Sensitive to Packaging, Synthesis and Metabolism

We first questioned histamine packaging with tetrabenazine, which is selective for VMAT2 (responsible for packaging in neurons). Tetrabenazine caused significant decreases in overall evoked histamine in both males and females highlighting the major role that VMAT2 plays in packaging neuronal histamine. This finding is in line with prior reports in zebrafish [32]. An additional agent with affinity for VMAT2 inhibition is reserpine., which also has affinity for VMAT1, but because VMAT1 is exclusively located in endocrine cells, utilizing this agent allows us to test VMAT2 inhibition in the brain [33]. Reserpine administration similarly decreased histamine release in both male and female mice. 

We next tested if histamine synthesis affected evocable brain histamine. Other groups have used FMH to successfully lower histamine [34,35,36] and here we utilized the same compound at doses previously described. Due to the extremely limited amount of the compound available to us (this compound is not currently commercially available and was custom synthesized for this study), we combined the sexes’ responses for FMH (20 mg kg^−1^) thus we are not able to comment on sex specific effects for this drug. However, our data are in good agreement with prior reports showing that FMH decreases histamine [36]. 

Finally, we targeted histamine metabolism. CNS histamine is metabolized exclusively by histamine N-methyltransferase which is located intracellularly; we utilized tacrine to inhibit this enzyme [37,38,39]. In male and female mice, tacrine caused a significant slowing of histamine reuptake. Because histamine N-methyltransferase is located intracellularly, inhibition of the enzyme results in higher systolic histamine levels, which manifests in the electrochemical signal as a slowed reuptake rate. This is because of the now higher concentration gradient that the transporters have to work against to clear histamine. A limitation of this experiment is that tacrine has additional affinity for blocking acetylcholine esterase which we acknowledge may confound our interpretations [39,40]. While it is well established that histamine modulates acetylcholine in the brain [41], the reverse notion is less explored. There is evidence that in mast cells in vitro, acetylcholine is a powerful histamine releaser [42] and one study found that acetylcholine modulates histamine release via muscarinic receptors in the hypothalamus of the rat [43]. Both studies posit that acetylcholine is a histamine releaser, therefore we cannot be certain that all the effect we observe here is due to histamine metabolism. Nonetheless, our data imply that histamine has similar packaging and synthesis/metabolism mechanisms to other common neurotransmitters such as dopamine and serotonin. Next, we studied histamine receptor pharmacology.

### 3.3. H3R Autoreceptors Differentiate Histaminergic Response in Male and Female Mice

There is a substantial body of literature documenting the autoregulatory role of the H3 receptor [16,17,31]. To test this in our system, we administered immepip, an H3R agonist, to cohorts of male and female mice. In both cases, we found that H3R agonism resulted in a significant decrease in evoked histamine, and modeling these data mathematically confirmed autoregulatory action via H3Rs. Next we turned to H3R antagonism; we previously utilized the antagonist thioperamide when developing histamine FSCV [13,24] and found dose dependent increases in evoked histamine release in male mice [13]. Here, we repeat that finding and our models again confirm H3R autoregulation. However, critically, an equivalent dose of thioperamide to female mice did not alter evoked histamine and our model did not require a change in autoregulation after this drug. This effect is likely not due to differences in receptor expression, since comparable H3 expression is found in male and female rats, ref. [44] or function, as evidenced by our experiments. Here, in a cohort of female mice, the effects of agonism could be reversed by thioperamide. Importantly evoked histamine levels were restored to control, but no higher, by thioperamide, not only validating the functionality of the receptors but also showing that a threshold level of evoked histamine cannot be surpassed in female mice by thioperamide. Another important aspect of this agonism/antagonism experiment is verification of thioperamide’s effects on H3Rs in this context since this agent also has affinity for H4Rs.

Therefore, a clear, intrinsic control is present in female mice that strictly regulates the levels of evoked histamine in the hypothalamus. This increased control may have evolutionary underpinnings as it is often thought that female animals exhibit more homeostatic control and that female hormones, estrogens and progesterones, have neuroprotective functions [45]. While histamine’s roles in bodily inflammation are well-established, less is known about histamine’s inflammatory functions in the brain.

We recently published work that showed rapid (5–10 min) increases in evoked hypothalamic histamine in mice upon systemic LPS injection, that we attributed to an inflammatory response [25]. Here, we repeated this experiment in a separate cohort of male mice and also observe an initial spike in evoked histamine from 5–20 min. Interestingly, from 20–30 min the evoked histamine begins to decline and then presents a bimodal increase until the end of the experiment. While initially we attributed the rapid spike to inflammation alone [25] we add here the hypothesis that the response may also mediate pain. LPS induced inflammatory pain is well-described [46,47]. In this model it is thought that LPS triggers the synthesis of proinflammatory cytokines and activation of tissue resident macrophages and neutrophils that release inflammatory mediators to regulate pain perception [47]. This process is well studied; however, it cannot account for the immediate (within minutes) effects seen with LPS [48,49]. An elegant study from Viania and colleagues showed that LPS caused a rapid, membrane delimited, excitation via transient receptor potential cation channels (TRPA1) [50]. TRPA1 links external irritant stimuli with nociceptor (pain receptor) activity. Here, we propose that histamine may be part of this signaling cascade (via the first histamine peak), while the second peak is ongoing inflammation due to more inflammatory processes being recruited. Histamine has been thought to play a role in pain previously and rapid physiological responses, while not processed due to anesthesia, remain intact [51,52].

Ferretti et al. suggested that stressor-induced increases in histamine release may be lower in females than males [21], given that we found tighter H3R control of histamine release in female mice, we repeated the LPS inflammation experiment in a cohort of female mice. We found that the LPS histamine response in female mice is heavily blunted, if not, totally compensated for. A small increase upon injection (10 min) may signal pain as we hypothesized above, however this response is significantly lower than in the male mice. There is long standing evidence that male and female mice process pain differently [53,54]. More recently, Mogil and colleagues have revealed that pain hypersensitivity mechanisms in female mice are dramatically different than in male mice and involve adaptive immune cells such as T-lymphocytes [55]. Thus, the differences in our histamine signal between male and female mice shortly after LPS injection are not surprising, and very informative of completely different signaling mechanisms that mediate pain perception between the sexes. Importantly, after this initial response, the histamine levels do not go above baseline, and in fact decrease. A large body of work has established that estradiol is a potent neuroprotective factor with roles at both the level of mitigating onset of disease/injury and reducing the pathological consequences of the disease/injury [56,57]. This hormone has been shown to be an important regulator in the hypothalamus [58]. H1R and the estrogen receptor alpha (ERα) mRNA are co-expressed in histaminergic neurons [58] and ERβ are expressed in the TMN [59]. The localization of estrogen receptors on histamine projections highlights the potential role estrogen plays in regulating immune response. Indeed, estrogen and progesterone have been shown to mitigate the acute inflammatory response to LPS exposure [60,61,62].

In sum, we find that evoked histamine in female mice is strictly governed, such that neither thioperamide nor LPS is able to significantly elevate evoked histamine in female mice. These findings may provide an interesting new avenue to explore in studies that investigate neuroprotection mechanism in females.

### 3.4. H1R Antagonist Modulates Serotonin Levels

Next, we looked at postsynaptic H1 and H2 receptor pharmacology. H1 receptor antagonists are clinically available as antihistamines [63], and H2 receptor antagonists are used clinically to reduce stomach acid production for chronic reflux [64], both are based on the notion of reducing the histamine signaling cascades that result in unwanted inflammatory responses.

Zolantidine (H2 receptor antagonist) administration did not significantly affect histamine release or reuptake, however an H1 receptor antagonist (DPH), in both male and female mice, resulted in histamine’s lifetime in the synapse to be prolonged. This effect may be due to slowed uptake by transporters (we and others recently showed that histamine is reuptaken by organic cation transporters) or prolonged histamine release by the presynaptic cell. Either scenario necessitates crosstalk between H1 receptors and the presynaptic cell’s transporters [65] or activity; there is precedence for this since similar effects have been observed with D2 receptor inhibition [66,67,68]. Our modeling pointed us towards crosstalk of activity of the presynaptic cell by the post-synaptic cell upon H1 activation. In an elegant 2000 review by Haas and colleagues [69], evidence was presented for H1 receptor modulation of presynaptic transmitter release via retrograde messengers such as arachidonic acid (AA) and nitric oxide (NO) [70,71,72]. In the review, the authors put forth this hypothesis of retrograde messengers, pointing out the proposed mechanism is yet to be experimentally demonstrated. We believe we have now provided compelling evidence for Haas’s hypothesis and present this retrograde messenger as **S** in our proposed model (Figure 4D). While our work cannot establish the identity of such a messenger, there is compelling evidence in the literature that endocannabinoids play important roles in retrograde signaling, either by DSI (depolarization-induced suppression of inhibition) or via DSE (depolarization-induced suppression of excitation) [73].

We thus present evidence for modulation of activity of histamine release via H1 receptors. The effect of this phenomenon (increased histamine lifetime in the synapse) is perhaps contrary to the intended mode of action of such a drug (classic antihistamine designed to stop histamine signaling). Because the H1 receptor is antagonized, presumably these increased histamine levels do not contribute to increased histamine signaling (as per the pharmacological intention). However, in the context of serotonin/histamine modulation, these findings may present another nuance; since we know that histamine inhibits serotonin release, what happens to serotonin if histamine signaling is prolonged?

**Figure 5:**
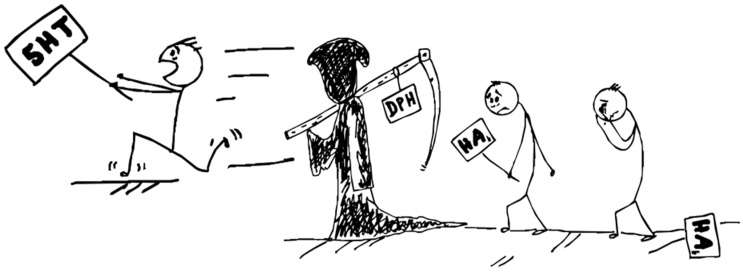
A comic strip illustration of DPH effects on histamine and serotonin.

We thus looked at the inhibition of serotonin by histamine before and after DPH and found a non-significant trend towards increased inhibition after DPH. We therefore, formally tested the notion that DPH affects serotonin by measuring ambient (minute to minute) serotonin levels in the CA2 region of the hippocampus in a separate cohort of mice and found that after a large, acute dose of DPH, serotonin levels rapidly, significantly fall. It is worth noting that this large DPH dose is clinically considered as ‘overdose’, (indeed DPH overdose symptoms are consistent with serotonin depletion (depression, anxiety, increased sleepiness) [74,75] and that acute, clinical doses of DPH are unlikely to have significant effects on serotonin. Additionally, DPH is a known antimuscarinic compound [76] (a muscarinic acetylcholine receptor antagonist) and thus at these high doses may induce anticholinergic syndrome, with many of the same presentations as DPH overdose. Nonetheless, this finding does highlight the fact that one should consider serotonin when investigating/designing pharmaceuticals for histamine. We illustrate this important idea with a comic figure in Figure 5, in appreciation of a comic figure from the 1990 Wada paper describing FMH pharmacology [35].

To sum our work, we performed an in-depth characterization of fast chemical histamine dynamics by pharmacologically targeting histamine synthesis, packaging, autoreceptor control of release, reuptake and metabolism. We presented two particularly meaningful, breakthrough aspects of histamine modulation. First, we found the differences in H3 regulation between the sexes, showing that in female mice evoked histamine could not be increased via H3 inhibition or an inflammatory stimulus. This led us to hypothesize that histamine may underlie neuroprotective mechanisms in female mice. Second, we found that high dose DPH rapidly decreased serotonin levels. While such high doses are considered overdose, this finding showcases how histaminergic targeting drugs have downstream effects on other modulators. This work highlights the sheer significance of better considering sex and the modulatory nuances of other neurotransmitters (such as serotonin) when studying/designing pharmaceuticals.

## 4. Materials and Methods

### 4.1. Chemicals and Reagents

All chemicals were used as received from the supplier. Diphenhydramine hydrochloride (20 mg kg^−1^ or 50 mg kg^−1^ Sigma Aldrich, St. Louis, MO, USA), zolantidine dimaleate (10 mg kg^−1^; Tocris, Minneapolis, MN, USA), immepip dihydrobromide (5 mg kg^−1^; Sigma Aldrich, St. Louis, MO, USA), thioperamide maleate (20 mg kg^−1^; Sigma Aldrich and Tocris), tacrine hydrochloride (2 mg kg^−1^; Tocris), and α-fluoromethylhistidine (20 mg kg^−1^; Toronto Research Chemicals, North York, ON, CAN) were all dissolved in sterile saline (0.9% NaCl solution, Mountainside Medical Equipment, NY, USA) at 5 mL kg^−1^. Reserpine (10 mg kg^−1^; Sigma Aldrich, St. Louis, MO, USA) was dissolved in 0.1% acetic acid (Sigma Aldrich, St. Louis, MO, USA) in sterile saline at 5 mL kg^−1^. Tetrabenazine (Sigma Aldrich) was dissolved in 10% DMSO (Sigma Aldrich, St. Louis, MO, USA) in sterile saline with 1 M HCl (10 µL mL^−1^ injection volume). All solutions were made fresh at the time of injection and all injections were given via intraperitoneal (*i.p*.) injection. Urethane (Sigma Aldrich, St. Louis, MO, USA) was dissolved in sterile saline as a 25% w/v solution and administered at 7 µL g^−1^.

### 4.2. Electrode Fabrication

All electrodes were made in house. A single carbon fiber was aspirated into a borosilicate capillary (0.6 mm × 0.4 mm × 10 cm; OD × ID × L) (A-M Systems, Sequim, WA, USA) and sealed under gravity and heat by a vertical pipette puller (Narishige, Amityville, NY, USA) to create two separate electrodes. The protruding fiber was trimmed under a light microscope to ~150 µm. An electrical connection was forged with the fiber through a stainless-steel connecting wire (Kauffman Engineering, Cornelius, OR, USA) and silver epoxy. Finally, a thin layer of Nafion (LQ-1105-MeOH), Ion Power, New Castle, DE, USA) was electrodeposited onto the fiber surface at 1 V for 30 s; the coated fiber was dried for 10 min at 70 °C [77].

### 4.3. Animals and Surgical Procedures

All animal procedures were in accordance with the regulations of the Institutional Animal Care and Use Committee (IACUC) at the University of South Carolina, accredited through the Association for Assessment and Accreditation of Laboratory Animal Care (AAALAC). Male and female C57BL/6J mice (The Jackson Laboratory) aged 6–12 weeks were used. Animals were group housed with ad libitum access to food and water and were kept on a 12 h light/dark cycle (0700/1900, on/off).

Stereotaxic surgery (David Kopf Instruments, Tujunga, CA, USA) followed induction of deep and sustained anesthesia from an intraperitoneal (i.p.) injection of urethane (described above). Mouse body temperature was maintained by thermal heating pad (Braintree Scientific, Braintree, MA, USA). All surgical coordinates were taken in reference to bregma [78]. A CFM was lowered into the posterior hypothalamus (AP: −2.5, ML: 0.5, DV: −5.5 to −5.6) and a stimulating electrode (insulated stainless-steel, d = 0.2 mm, untwisted; Plastics One, Roanoke, VA, USA) was placed into the medial forebrain bundle (AP: −1.1, ML: +1.1, DV: −5.0) [13].

The Ag/AgCl reference electrode, created by chloridizing a polished silver wire (15 s in 1M HCl at 5 V), was placed in the contralateral hemisphere. All agents were given via i.p. injection at doses determined suitable from previous studies. Vehicle solutions were administered (5 mL kg^−1^) to 30 min between control and drug files to determine impact. Here, vehicle injections did not significantly change the evoked release in males or females. Some agents were not soluble in saline. Reserpine was dissolved in 0.1% acetic acid (AcOH) in saline, tetrabenazine required 10% DMSO in saline with 1 M HCl (10 µL mL^−1^). The post-drug files show the maximal effect within 1 h per drug.

To analyze sex and estrous cycle, control histamine and serotonin data were pooled. Due to the sensitivity of the measurements being made, we are unable to determine the estrous cycle stage prior to the experiment as we have observed in previous animals that doing so influences release and reuptake characteristics. For cycle determination, vaginal lavage was performed following the conclusion of data collection. Briefly, approximately 10 µL of sterile saline was administered and quickly removed from the vagina and then visualized under low power light microscope to determine estrous cycle stage via cytological examination [79].

### 4.4. Data Collection and Analysis

FSCV was performed on anesthetized mice using a Chem-Clamp potentiostat (Dagan Corporation, Minneapolis, MN, USA), custom built hardware interfaced with PCIe 6341 & PCI 6221 DAC/ADC cards (National Instruments, Austin, TX, USA), and a Pine Research headstage (Pine Research Instruments, Durham, NC, USA). WCCV 3.06 software (Knowmad Technologies LLC, Tucson, AZ, USA) was used for data analysis. The histamine waveform (−0.5 to −0.7 to +1.1 to −0.5 V at 600 V s^−1^) was applied at 60 Hz for 10 min, then at 10 Hz for 10 min prior to data collection at 10 Hz. Histamine was evoked via biphasic stimulation applied through a linear constant current stimulus isolator (NL800A Neurolog, Digitimer North America LLC, Fort Lauderdale, FL, USA) with stimulations at 60 Hz, 360 µA, 2 ms in width, and 2 s in length.

FSCAV was performed to measure ambient concentrations of serotonin using the same hardware as described above for FSCV. The serotonin waveform was initially applied at a frequency of 100 Hz for 2 s to minimize adsorption to the carbon fiber surface. After that, a period of controlled adsorption (10 s at 0.2 V) was applied to allow serotonin to preconcentrate at the carbon surface. Finally, the waveform was reapplied at 100 Hz and the third CV was selected to estimate serotonin extracellular concentration [80]. After 30 min of stable control files, DPH (i.p. injection, 50 mg kg^−1^) was administered and files were taken for an additional 60 min. Charge under the serotonin Faradaic peak was detected and integrated using custom-designed algorithms in The Analysis Kid [81]. Charge was converted to extracellular serotonin concentration using calibration factors obtained from calibrations specific for each electrode.

Data were collected and filtered on WCCV software (Butterworth, 3 kHz low pass filter). Four control evoked files 10 min apart were averaged for the control evoked histamine signal after which a compound was administered and data were collected every 10 min for 2 h. For the immepip-thioperamide experiments, data were collected for 60 min followed by administration of thioperamide immediately after the 60 min file was collected. Files were then collected for an additional 60 min. Current was converted to concentration through calibration factors for both histamine (2.8 µM nA^−1^) and serotonin (11 µM nA^−1^) [24]. A large voltage (~10 V; ~2 min) was used to lesion the tissue surrounding the CFM.

### 4.5. Mathematical Modeling of the Experimental Data

For all of our modeling experiments, we incorporated data on the H3 receptor into the model of histamine synthesis, release, reuptake and control by H3 autoreceptor that we have previously published [13,71]. In all experiments, the 0 on the y-axis represents the baseline concentration of histamine in the extracellular space. All experimental curves are plotted relative to baseline. Similarly, in the mathematical model we adjusted parameters so that the baseline concentration of extracellular histamine was 5.32 µM in all simulations except female-thioperamide. In that case, the extracellular concentration descended approximately 8 µM below baseline so the baseline concentration must be higher than 8 µM. We adjusted the reuptake parameter so that the baseline concentration was 14.6 µM. It makes sense that the baseline concentration rises after thioperamide administration because thioperamide is an H3 antagonist. The results of simulation are plotted in each case relative to baseline.

Calculations of solution curves were performed in MATLAB using the differential equations solver ODE15s. The complete model is described in [70] and the code is available from the authors by request. For all experiments we first made small adjustments in parameters so that the model solution matched the experimental control curve. We then investigated what change of parameters made the solution curve match the experimental post-drug curve.

### 4.6. Statistical Analyses

Average control response was generated from four current vs. time traces per animal and averaged to create an overall group average. To determine the t_1/2_, a code was custom written in Excel to fit the reuptake component of the curve and calculate the time taken to reach half the maximum amplitude. Exclusion criteria were based on outliers (via Grubbs test) and animals that did not survive the experimental paradigm. Standard error of the mean (SEM) was calculated using the average response of each animal (*n* = # animals). Significance between two points was determined by 2-tailed paired *t*-test or independent test and taken as *p* < 0.05. For non-normally distributed data (via Shapiro–Wilk test), the Kruskal–Wallis H test was used to determine significance and taken as *p* < 0.05. All error bars represent the standard error of the mean (SEM). Differences between more than two independent groups were tested using ANOVA and post hoc multiple comparisons *t*-test. Differences in slopes of time series were tested using ANCOVA and post hoc multiple comparisons *t*-test.

### 4.7. Post Experimental Histological Analysis

Tissue immediately around the CFM was lesioned by applying 10V to the CFM for 20 s. Lesioned brains (previously stored in 4% paraformaldehyde and transferred to 30% sucrose 24 hr prior to analysis) were flash frozen and mounted onto a cutting slide. 25 μm tissue slices were collected and visualized under light microscope. The tissue lesion coordinates (A/P and D/V) were recorded in reference to bregma to determine brain region [78] and are shown in Figure 1D.

## Data Availability

Data are available upon request.
